# Characteristics and outcomes of ICU patients with bacterial catheter-associated urinary tract infection

**DOI:** 10.1371/journal.pone.0358796

**Published:** 2026-09-25

**Authors:** Mateusz Bartoszewicz, Marta Krysik, Sławomir Lech Czaban, Jerzy Robert Ładny

**Affiliations:** 1 Department of Anaesthesiology and Intensive Care, Medical University of Bialystok, Bialystok, Poland; 2 Medical University of Bialystok, Bialystok, Poland; 3 Department of Emergency Medicine, Medical University of Bialystok, Bialystok, Poland; AIIMS: All India Institute of Medical Sciences, INDIA

## Abstract

**Background:**

Catheter-associated urinary tract infection (CAUTI) is a common device-associated complication in intensive care units (ICUs).

**Objective:**

To characterize bacterial CAUTI in an ICU cohort and identify independent associations with demographic and clinical characteristics available at or before ICU admission.

**Methods:**

This single-center retrospective cohort included 3326 index ICU admissions from January 2017 through June 2023. After exclusion of 95 fungal-only clinically classified cases, 3231 records remained. Bacterial CAUTI required compatible clinical findings, at least 48 hours of catheter exposure, and ≥10³ CFU/mL of one or two bacterial species. All patients were catheterized throughout the observed ICU stay. The primary logistic model included only admission characteristics; ICU stay/catheter duration was descriptive because exact CAUTI onset dates were unavailable. Skewed biomarkers were summarized with medians, interquartile ranges, and Mann-Whitney tests.

**Results:**

Bacterial CAUTI occurred in 301 patients (9.3%; 95% CI 8.4%−10.4%). Mean ICU stay/catheter duration was 28.3 versus 13.6 days (difference 14.8 days; 95% CI 11.7–17.8), but duration was not interpreted as an independent risk estimate. Mortality was 49.2% versus 45.9% (risk difference 3.2 percentage points; 95% CI −2.7 to 9.1), and bacterial CAUTI was not associated with death after adjustment for admission characteristics (OR 0.95; 95% CI 0.75–1.22). Female sex (OR 1.48; 95% CI 1.15–1.90), obesity (OR 1.62; 95% CI 1.20–2.19), and COVID-19 (OR 2.16; 95% CI 1.55–3.00) were independently associated. At least one resistance phenotype was recorded in 200 patients (66.4%).

**Conclusions:**

Bacterial CAUTI was associated with a distinct admission profile and longer observed ICU stay/catheter exposure, although causal direction could not be determined. It was not associated with adjusted in-hospital mortality.

## Introduction

Healthcare-associated infections remain an important source of morbidity in intensive care units (ICUs), where critical illness, immune dysfunction, antimicrobial exposure, and invasive devices converge [1–4]. Catheter-associated urinary tract infection (CAUTI) is clinically relevant because indwelling bladder catheters are frequently required for hourly urine-output measurement and fluid-balance management. These infections increase diagnostic testing, antimicrobial exposure, and the risk of secondary bloodstream infection [5,6].

An indwelling urethral catheter disrupts normal urinary defenses, promotes ascending microbial migration, and provides a surface for biofilm formation. Risk increases with cumulative device exposure; prevention guidance therefore emphasizes appropriate indications, aseptic insertion, maintenance of a closed drainage system, and prompt removal when continuous monitoring is no longer required [5–7].

The microbiology of catheter-associated infection in the ICU differs from uncomplicated community-acquired urinary infection. Enterococcus, Klebsiella, Pseudomonas, Acinetobacter, Staphylococcus, and other healthcare-associated organisms may predominate after prolonged hospitalization and broad-spectrum antimicrobial treatment. Extended-spectrum beta-lactamase (ESBL), metallo-beta-lactamase (MBL), vancomycin resistance, and high-level aminoglycoside resistance (HLAR) can substantially restrict treatment options [8–12].

The COVID-19 pandemic altered ICU case mix and device exposure. Severe SARS-CoV-2 infection frequently required prolonged mechanical ventilation, deep sedation, prone positioning, corticosteroids, and extended catheterization. A previous publication from our center examined CAUTI in 201 patients with COVID-19 admitted between March 2020 and July 2021 [13]. The broader cohort analyzed here includes that previously reported subset but addresses bacterial CAUTI across all ICU diagnoses over more than six years.

The primary objective was to estimate the proportion of ICU patients who developed bacterial CAUTI and identify independent associations with demographic and clinical characteristics available at or before ICU admission. Secondary objectives were to quantify mortality and co-occurring device-associated infections, describe ICU length of stay/observed catheter exposure without assigning causal direction, compare TISS-28 interventions after normalization to observation time, and describe local bacterial and antimicrobial resistance patterns.

## Materials and methods

### Study design, setting, and reporting

This single-center retrospective cohort study was conducted in the ICU of the University Clinical Hospital in Bialystok, Poland. The source period extended from January 1, 2017, through June 1, 2023. Reporting follows the Strengthening the Reporting of Observational Studies in Epidemiology (STROBE) recommendations [14].

### Study population and index admission

The source cohort comprised 3326 index ICU admissions. Only the first eligible ICU admission for each patient was retained. A secondary ICU hospitalization was defined as any later ICU admission after discharge from the index ICU, whether during the same hospital episode or a subsequent hospitalization; these later admissions were excluded to avoid repeated observations from the same patient.

### Relationship to prior publications

**Fig 1:**
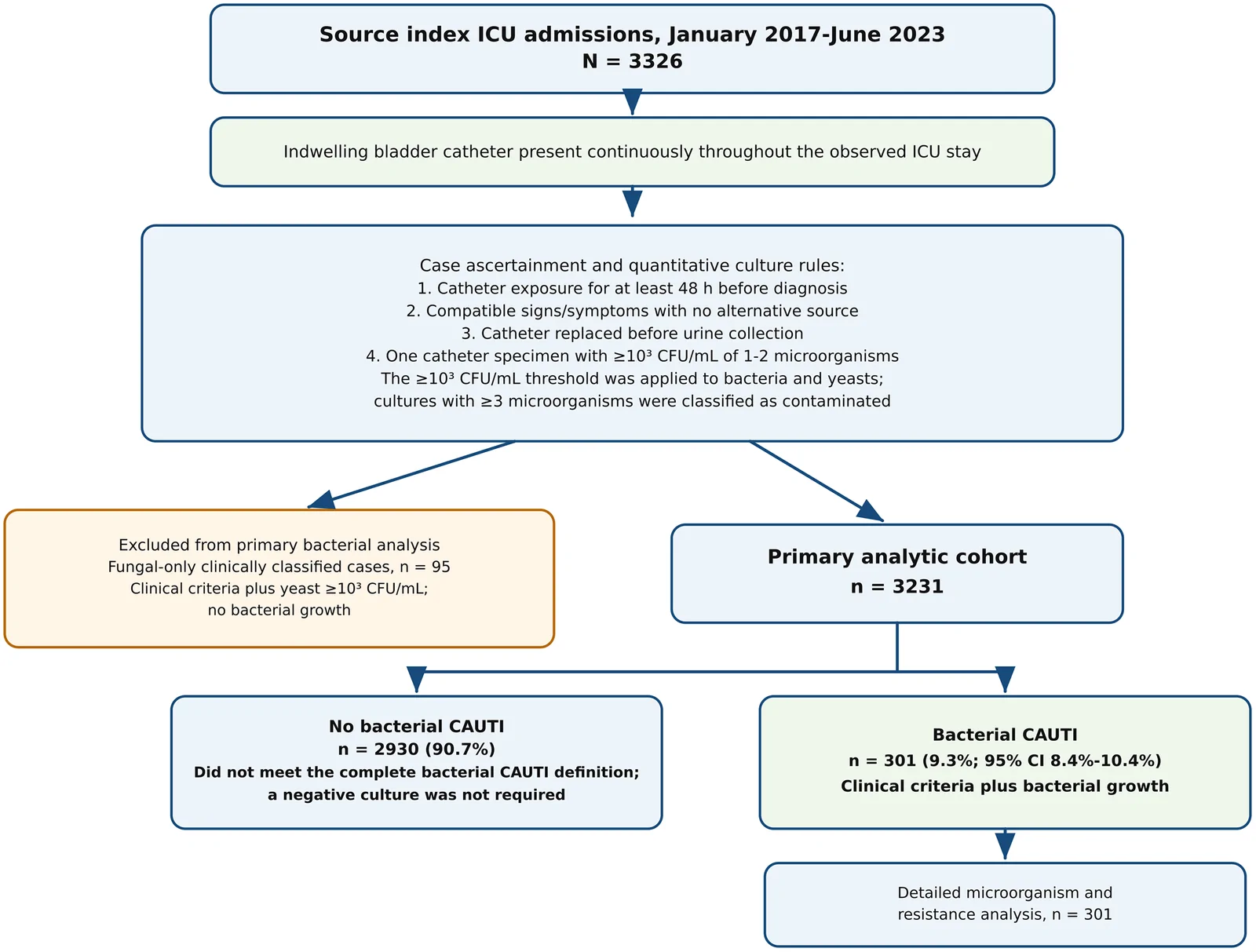
Study cohort, revised exclusions, and bacterial CAUTI diagnostic criteria.

The database includes the 201-patient COVID-19 cohort previously reported by Dąbrowska et al. [13], which was restricted to COVID-19 admissions from March 2020 through July 2021. To avoid duplicate reporting, that publication is identified as an overlapping subgroup. The present revised analysis uses a bacterial-only primary outcome across the full source cohort, excludes 95 fungal-only records identified in the broader dataset, includes 301 bacterial CAUTI cases and 2930 comparison records, extends follow-up through June 2023, normalizes TISS-28 interventions to person-time, and evaluates multivariable associations across ICU diagnoses. All COVID-19 diagnoses occurred from March 2020 onward. The cohort flow and bacterial classification are summarized in Fig 1.

The figure shows the source cohort, continuous ICU catheter exposure, the complete clinical and microbiological criteria, exclusion of fungal-only clinically classified cases, and the revised primary bacterial CAUTI groups. The no-bacterial-CAUTI group was not defined by a negative culture. CAUTI, catheter-associated urinary tract infection; CFU, colony-forming units; CI, confidence interval; ICU, intensive care unit.

### Primary and secondary outcomes

The primary outcome was bacterial CAUTI during the index ICU admission. Secondary outcomes were in-hospital death, ICU length of stay/observed ICU catheter exposure, bacterial bloodstream infection (BSI), ventilator-associated pneumonia (VAP), normalized TISS-28 intervention rates, and patient-level bacterial organism and resistance-phenotype distributions. BSI and VAP were recorded if documented at any time during the index ICU stay; event dates were unavailable, so temporal ordering relative to CAUTI could not be established.

### Clinical and microbiological case definition

All patients had an indwelling bladder catheter continuously throughout the observed ICU stay. Urine cultures were obtained when clinical features suggested urinary tract infection. Bacterial CAUTI required catheter exposure for at least 48 hours before diagnosis; compatible findings such as fever, suprapubic or costovertebral-angle discomfort, altered mental status, hypotension, or systemic inflammatory response without another identified source; and a positive catheter urine culture. The catheter was replaced before sample collection to reduce contamination by established biofilm. A positive bacterial culture was defined as ≥10³ colony-forming units (CFU)/mL of one or two bacterial species in a single catheter specimen; cultures with three or more microorganisms were classified as contaminated. Microbiological specimens were obtained before antimicrobial treatment whenever clinically feasible.

The same quantitative threshold of ≥10³ CFU/mL was applied to yeast growth in the source classification. For the revised primary bacterial analysis, 95 clinically classified cases with fungal growth but no bacterial growth were excluded rather than reclassified as non-CAUTI. Patients with bacterial growth remained in the bacterial CAUTI group when concurrent fungal urine growth was also recorded.

### Urine culture, identification, and antimicrobial susceptibility testing

For catheterized patients, approximately 5 mL of urine was aspirated aseptically from the disinfected sampling port of the closed Foley-catheter system after catheter replacement. Specimens were transported to the laboratory within 2 hours. When immediate transport was not possible, specimens were refrigerated at 4°C and cultured within 4 hours. Quantitative culture used a sterile calibrated 1-µL loop (0.001 mL), so one colony represented 10³ CFU/mL. Plates were incubated and read after 18–24 hours; incubation was extended to 48 hours when clinically indicated or after prior antimicrobial exposure, and fungal cultures were incubated for at least 48 hours.

### Blood cultures and bacteremic CAUTI

The retrospective extract did not document protocol-mandated blood-culture collection at every CAUTI diagnosis. Blood cultures were therefore treated as clinically indicated rather than systematic. BSI denotes any bacterial bloodstream infection during the ICU stay; bacteremic CAUTI could not be distinguished reliably from coincident or secondary BSI.

### Data collection and variables

Demographic characteristics, comorbidities, admission laboratory results, ICU interventions, and outcomes were extracted from electronic records. Covariates for the primary multivariable model were selected on clinical grounds and were restricted to characteristics available at or before ICU admission: age, sex, obesity, COVID-19, diabetes mellitus, heart and/or respiratory failure, atrial fibrillation, and acute myocardial infarction or ischemic stroke. Obesity was classified from BMI-derived or documented obesity fields. Systemic steroid therapy was retained as a descriptive variable but was not included in the primary model because its timing relative to CAUTI was unavailable. Admission biomarkers were not included in the primary model because of substantial missingness.

### Catheter exposure and normalized TISS-28 analysis

Because every patient had an indwelling bladder catheter continuously from ICU admission until ICU discharge or death, observed ICU catheter exposure equaled ICU length of stay. Pre-ICU catheter exposure and exact CAUTI onset times were not available for the full cohort. ICU length of stay/catheter duration could therefore include time both before and after CAUTI and was not entered into the primary logistic regression. It was summarized descriptively, and a crude incidence estimate was calculated per 1000 observed ICU catheter-days among records with complete ICU length of stay. A time-dependent or competing-risk analysis could not be performed without reliable CAUTI onset dates. TISS-28 items were expressed as intervention-days per 100 observed TISS days. Rate ratios were estimated with Poisson regression using log(observation days) as an offset and heteroskedasticity-robust standard errors.

### Statistical analysis

Continuous variables are reported as mean (standard deviation). Because CRP, procalcitonin, and lactate showed marked right-skew, these biomarkers are also reported as median [interquartile range], with two-sided Mann-Whitney U tests presented alongside Welch t tests. Categorical variables are reported as number (percentage). The bacterial CAUTI proportion was accompanied by a Wilson 95% confidence interval (CI). Key continuous differences used Welch 95% CIs; key categorical outcomes used Newcombe 95% CIs for risk differences. The primary multivariable logistic regression estimated adjusted odds ratios (ORs) for bacterial CAUTI using only admission characteristics and heteroskedasticity-robust standard errors. ICU length of stay/catheter duration, TISS-28 intensity, systemic steroid therapy, BSI, and VAP were excluded from this model because they accrued after admission or lacked reliable temporal ordering relative to CAUTI. A separate admission-characteristic-adjusted logistic model assessed the association between bacterial CAUTI and in-hospital death. Complete-case analysis was used. Biomarker comparisons were exploratory and were not adjusted for multiplicity. Statistical significance was defined as two-sided p < 0.05. Analyses used Python 3.13, pandas 2.2.3, SciPy 1.17.0, statsmodels 0.14.6, and scikit-learn 1.8.0.

### Sample size and precision

No a priori sample-size calculation was performed because all eligible index admissions in the source period were included. With 301 bacterial CAUTI and 2930 comparison patients, the available sample had approximately 80% power at a two-sided alpha of 0.05 to detect an absolute mortality difference of about 8.5 percentage points, assuming 45.9% mortality in the comparison group. Interpretation emphasizes effect estimates and 95% CIs rather than post hoc significance alone.

### Ethics

The study was conducted in accordance with the Declaration of Helsinki. The Bioethics Committee of the Medical University of Bialystok, Poland, confirmed that this retrospective study did not constitute a medical experiment and did not require prospective ethics committee approval (protocol APK.002.82.2024; February 22, 2024). The requirement for informed consent was waived because routinely collected retrospective data were used.

## Results

### Cohort and bacterial CAUTI occurrence

The source cohort included 3326 index ICU admissions. After exclusion of 95 fungal-only clinically classified cases, the primary analytic cohort included 3231 records. Bacterial CAUTI occurred in 301 patients (9.3%; 95% CI 8.4%−10.4%); 2930 patients did not meet the complete bacterial CAUTI definition (Fig 1).

### Patient characteristics and admission laboratory variables

Patients with bacterial CAUTI were more frequently female or obese and had higher frequencies of COVID-19, atrial fibrillation, and systemic steroid treatment; they were also older on unadjusted comparison. For the right-skewed biomarkers, CRP was lower in the bacterial CAUTI group by both Welch and Mann-Whitney testing (median 99.6 [IQR 53.9–170.4] vs 119.2 [62.4–193.0] mg/L; Mann-Whitney p = 0.010). Procalcitonin means did not differ by Welch testing (p = 0.270), whereas the median was lower in the bacterial CAUTI group (0.73 [0.23–3.04] vs 1.13 [0.26–5.01] ng/mL; Mann-Whitney p = 0.029). Lactate means differed by Welch testing (p < 0.001), but medians were nearly identical (1.89 [1.35–2.54] vs 1.80 [1.27–2.80] mmol/L; Mann-Whitney p = 0.927), indicating that the mean difference was driven by upper-tail values rather than a general shift in the distribution. WBC did not differ by Welch testing, and glucose was higher in the bacterial CAUTI group (Table 1). BMI and admission biomarkers had substantial variable-specific missingness (Table C in S1 File).

**Table 1:** Patient characteristics and admission laboratory variables according to bacterial CAUTI status.

Variable	No bacterial CAUTI(n = 2930)	Bacterial CAUTI(n = 301)	Total(n = 3231)	p value(s)
Age, years	63.2 (16.6)	65.8 (16.1)	63.5 (16.6)	0.008
Female sex	1077 (36.8)	144 (47.8)	1221 (37.8)	<0.001
BMI, kg/m²	27.9 (7.0)	29.4 (7.0)	28.1 (7.0)	0.006
Obesity (BMI-based or documented)	592 (20.2)	87 (28.9)	679 (21.0)	<0.001
COVID-19	296 (10.1)	55 (18.3)	351 (10.9)	<0.001
Diabetes mellitus	495 (16.9)	56 (18.6)	551 (17.1)	0.452
Atrial fibrillation	343 (11.7)	52 (17.3)	395 (12.2)	0.005
Heart and/or respiratory failure	1155 (39.4)	126 (41.9)	1281 (39.6)	0.410
Acute myocardial infarction or ischemic stroke	113 (3.9)	17 (5.6)	130 (4.0)	0.132
Systemic steroid therapy	771 (26.3)	99 (32.9)	870 (26.9)	0.014
CRP at admission, mg/L	137.9 (100.5)119.2 [62.4-193.0]	121.2 (92.3)99.6 [53.9-170.4]	135.5 (99.6)116.9 [61.1-189.1]	0.008 / 0.010
WBC at admission, × 10³/µL	14.3 (11.4)	14.1 (16.8)	14.3 (12.2)	0.823
Procalcitonin at admission, ng/mL	6.9 (14.4)1.13 [0.26-5.01]	5.8 (13.7)0.73 [0.23-3.04]	6.7 (14.3)1.03 [0.25-4.68]	0.270 / 0.029
Glucose at admission, mg/dL	152.6 (48.3)	165.3 (53.3)	153.9 (49.0)	0.010
Lactate at admission, mmol/L	3.2 (4.1)1.80 [1.27-2.80]	2.4 (2.0)1.89 [1.35-2.54]	3.1 (3.9)1.80 [1.27-2.75]	<0.001 / 0.927

Values are mean (SD) or n (%). For CRP, procalcitonin, and lactate, the first line is mean (SD), the second line is median [IQR], and p values are reported as Welch t test / Mann-Whitney U test. Percentages use the group denominator. Variable-specific available totals were: age 3170, BMI 1945, CRP 1829, WBC 2364, procalcitonin 1627, glucose 1254, and lactate 1251. Chi-square or Fisher exact tests were used for categorical variables. Biomarker comparisons were exploratory and were not adjusted for multiplicity. CAUTI, catheter-associated urinary tract infection; CRP, C-reactive protein; IQR, interquartile range; WBC, white blood cell count.

### Clinical outcomes and catheter exposure

Mean observed ICU stay/catheter duration was 28.3 days in the bacterial CAUTI group and 13.6 days in the comparison group (difference 14.8 days; 95% CI 11.7–17.8; p < 0.001). Corresponding medians were 21 days (IQR 11–38) and 8 days (IQR 3–18). Among 3187 records with complete exposure data, 47713 observed ICU catheter-days and 301 bacterial CAUTI events yielded a crude incidence of 6.31 per 1000 observed ICU catheter-days. Because duration included time after CAUTI onset for affected patients and exact onset dates were unavailable, these comparisons are descriptive and do not estimate a causal effect of catheter duration.

In-hospital mortality was 49.2% with bacterial CAUTI and 45.9% without bacterial CAUTI (risk difference 3.2 percentage points; 95% CI −2.7 to 9.1; p = 0.284). In the admission-characteristic-adjusted mortality model, bacterial CAUTI was not associated with death (adjusted OR 0.95; 95% CI 0.75–1.22; p = 0.708). BSI and VAP were substantially more frequent among bacterial CAUTI patients, although temporal ordering could not be determined (Table 2).

**Table 2:** Key unadjusted clinical outcomes with 95% confidence intervals.

Outcome	No bacterial CAUTI	Bacterial CAUTI	Unadjusted difference(95% CI)	p value
ICU length of stay / observed ICU catheter exposure, days	13.58 (23.17)	28.33 (25.91)	14.75 (11.70 to 17.81)	<0.001
TISS-28 observation days	14.69 (16.02)	32.07 (27.40)	17.38 (14.22 to 20.54)	<0.001
Daily TISS intensity, item-days per observed day	12.69 (1.43)	12.85 (0.89)	0.17 (0.05 to 0.28)	0.004
In-hospital death	1346 (45.9)	148 (49.2)	3.2 pp (−2.7 to 9.1)	0.284
Bacterial bloodstream infection	272 (9.3)	86 (28.6)	19.3 pp (14.4 to 24.7)	<0.001
Ventilator-associated pneumonia	645 (22.0)	221 (73.4)	51.4 pp (45.9 to 56.3)	<0.001

Continuous outcomes are mean (SD) with Welch mean differences. Categorical outcomes are n (%) with Newcombe risk differences in percentage points (pp). ICU length of stay and observed ICU catheter exposure are identical because all patients were catheterized continuously throughout the observed ICU stay. BSI and VAP could precede or follow CAUTI because event dates were unavailable.

### Multivariable associations based on admission characteristics

The revised primary complete-case model included 3170 patients and all 301 bacterial CAUTI events. Female sex, obesity, and COVID-19 were independently associated with bacterial CAUTI. Age, diabetes, heart and/or respiratory failure, atrial fibrillation, and acute myocardial infarction or ischemic stroke were not independently associated (Table 3). ICU length of stay/catheter duration and other post-admission variables were not included. The model area under the receiver-operating characteristic curve was 0.620; the maximum non-intercept variance inflation factor was 1.18.

**Table 3:** Multivariable logistic regression for bacterial CAUTI occurrence using admission characteristics (n = 3170; 301 events).

Covariate	Adjusted OR	95% CI	p value
Age, per 10 years	1.05	0.97-1.14	0.252
Female sex	1.48	1.15-1.90	0.002
Obesity	1.62	1.20-2.19	0.001
COVID-19	2.16	1.55-3.00	<0.001
Diabetes mellitus	0.88	0.64-1.20	0.410
Heart and/or respiratory failure	1.03	0.78-1.37	0.830
Atrial fibrillation	1.36	0.97-1.92	0.076
Acute myocardial infarction or ischemic stroke	1.42	0.83-2.44	0.201

Heteroskedasticity-robust standard errors were used. The model was restricted to demographic and clinical characteristics available at or before ICU admission. ICU length of stay/observed catheter exposure, TISS-28 intensity, systemic steroid therapy, BSI, and VAP were excluded because they accrued after admission or lacked reliable temporal ordering relative to CAUTI. CAUTI, catheter-associated urinary tract infection; CI, confidence interval; OR, odds ratio.

### TISS-28 person-time results

Cumulative TISS intervention-days were higher among bacterial CAUTI patients because their observation time was substantially longer. After normalization, overall daily TISS intensity differed by 0.17 item-days per observed day (95% CI 0.05–0.28). Respiratory physiotherapy and enteral nutrition remained more frequent per observed day, whereas several other large cumulative differences attenuated or reversed. Selected normalized rates are shown in Table 4; all TISS-28 items are reported in Table A in S1 File.

**Table 4:** Selected TISS-28 intervention rates normalized to observed ICU days.

Intervention	No bacterial CAUTIper 100 days	Bacterial CAUTIper 100 days	Rate ratio(95% CI)	p value
Mechanical ventilation	79.1	81.2	1.03 (0.99-1.06)	0.136
Respiratory physiotherapy	57.4	69.7	1.21 (1.10-1.35)	<0.001
Single vasoactive drug	43.4	45.8	1.06 (0.96-1.16)	0.249
Central venous catheter	96.4	97.9	1.02 (1.00-1.03)	0.085
Renal replacement therapy	13.7	12.9	0.94 (0.71-1.25)	0.672
Quantitative urine-output monitoring	98.9	99.2	1.00 (1.00-1.01)	0.277
Parenteral nutrition	45.0	37.9	0.84 (0.73-0.98)	0.024
Enteral nutrition	68.3	73.7	1.08 (1.02-1.14)	0.006
Procedures outside the ICU	4.9	4.4	0.90 (0.73-1.10)	0.302

Rates are aggregate intervention-days per 100 TISS observation-days. Rate ratios were estimated with Poisson models using log(observation days) as an offset and robust standard errors. Full results are in Table A in S1 File.

### COVID-19 sensitivity analysis

All COVID-19 diagnoses occurred from March 2020 onward. Bacterial CAUTI occurred in 55/351 patients with COVID-19 (15.7%) and 246/2880 patients without COVID-19 (8.5%), an absolute difference of 7.1 percentage points (95% CI 3.5–11.4) and a risk ratio of 1.83 (95% CI 1.40–2.40). COVID-19 remained associated in the multivariable model. A calendar-era comparison of pre-pandemic versus pandemic non-COVID admissions could not be reconstructed because exact dates were not retained in the source analytical data.

### Microbiology and resistance

The detailed UTI microbiology analysis was restricted to 301 bacterial CAUTI records. Concurrent fungal urine growth was recorded in 54 patients (17.9%), who remained in the bacterial analysis because bacterial growth was also present. Organism-group variables were patient-level and non-mutually exclusive. Enterococcus, Klebsiella, Staphylococcus, Escherichia, and Acinetobacter were the most frequent recorded bacterial groups (Table 5).

**Table 5:** Recorded bacterial groups and resistance phenotypes among 301 bacterial CAUTI patients.

Bacterial group	n (%)	Resistance phenotype	n (%)
Enterococcus	138 (45.8)	HLAR	66 (21.9)
Klebsiella	102 (33.9)	ESBL	63 (20.9)
Staphylococcus	95 (31.6)	MDR	57 (18.9)
Escherichia	70 (23.3)	MBL	53 (17.6)
Acinetobacter	69 (22.9)	VRE	36 (12.0)
Pseudomonas	55 (18.3)	NDM	23 (7.6)
Proteus	32 (10.6)	MLSBI	11 (3.7)
Streptococcus	23 (7.6)	MRSA	7 (2.3)
Enterobacter	18 (6.0)	MLSBK	6 (2.0)
Stenotrophomonas	18 (6.0)	VIM	3 (1.0)
Coagulase-Negative Staphylococcus	13 (4.3)	KPC	2 (0.7)
Corynebacterium	12 (4.0)	OXA-48	1 (0.3)
Serratia	11 (3.7)		

Variables are patient-level and non-mutually exclusive. Percentages use 301 bacterial CAUTI patients as the denominator. W-OPOR/W-OPORNY was translated as MDR. OVA-48 was corrected to OXA-48. MSS was excluded because it denotes susceptibility. MRR was excluded because it was an unresolved local code and no validated MarR molecular assay was present. ESBL, extended-spectrum beta-lactamase; HLAR, high-level aminoglycoside resistance; MBL, metallo-beta-lactamase; MRSA, methicillin-resistant Staphylococcus aureus; NDM, New Delhi metallo-beta-lactamase; VRE, vancomycin-resistant Enterococcus.

At least one resistance phenotype was recorded in 200 patients (66.4%); 90 (29.9%) had at least two, 28 (9.3%) at least three, and 8 (2.7%) at least four. The most frequent pairwise co-occurrences were NDM with MBL (23), MBL with MDR/W-OPOR (16), ESBL with HLAR (15), ESBL with MDR/W-OPOR (14), and VRE with MDR/W-OPOR (11). NDM is an MBL subtype, so NDM-MBL co-recording partly reflects hierarchical coding.

## Discussion

After exclusion of fungal-only clinically classified cases, bacterial CAUTI occurred in 9.3% of the revised cohort. Bacterial CAUTI identified a population with substantially longer observed ICU stay/catheter exposure and a high burden of VAP and BSI. In the model restricted to admission characteristics, female sex, obesity, and COVID-19 remained independently associated. The unadjusted mortality difference was imprecise and crossed the null, and bacterial CAUTI was not associated with death after adjustment for admission characteristics.

The large difference in ICU length of stay/observed catheter duration is clinically relevant but cannot be interpreted as an independent risk estimate. Duration accrued after ICU admission and, for patients with CAUTI, could include both pre-infection time at risk and post-infection prolongation of care. Entering log2(days+1) into a standard logistic model would therefore condition on a post-admission variable that may partly be a consequence of CAUTI, creating reverse-causation and overadjustment bias. We removed ICU length of stay/catheter duration and daily TISS intensity from the primary multivariable model rather than interpreting the previous adjusted OR of 1.92. Exact CAUTI onset dates were unavailable, precluding a time-dependent Cox or competing-risk analysis. Duration is retained only as a descriptive time-at-risk measure, and the direction and magnitude of any causal relationship remain unresolved.

The incidence estimate of 6.31 bacterial CAUTI events per 1000 observed ICU catheter-days is lower than the previously reported 15.8 per 1000 catheter-days in the overlapping COVID-19 cohort [13]. The estimates are not directly interchangeable because the earlier study was restricted to severe COVID-19, whereas the present analysis covers all ICU diagnoses and applies a bacterial-only primary outcome after excluding fungal-only records from the broader source dataset. Explicit disclosure of the overlap prevents presentation of the earlier disease-specific result as if it were newly derived.

Person-time normalization materially changed interpretation of the TISS-28 findings. Cumulative intervention-days were much higher among bacterial CAUTI patients, but much of that difference reflected approximately twice as many observed days. Overall daily TISS intensity differed only modestly. Respiratory physiotherapy and enteral nutrition remained more frequent per observed day, while several other cumulative differences attenuated or reversed. This analysis avoids treating longer hospitalization as equivalent to uniformly greater daily care intensity.

The high frequency of Enterococcus is plausible in a prolonged, catheterized, and antibiotic-exposed ICU population. Enterococci adhere to catheter-associated biofilm, tolerate environmental stress, and may be selected by broad-spectrum antibacterial treatment [6,15,16]. Local colonization pressure and repeated sampling may also contribute. Because organism variables were non-mutually exclusive patient-level indicators and genomic typing was not performed, the data cannot distinguish independent episodes, persistent colonization, repeated isolation, or clonal transmission.

The revised analysis applies a bacterial case definition. The ≥ 10³ CFU/mL threshold was applied to yeasts in the source classification, but 95 fungal-only clinically classified cases were excluded to avoid combining candiduria or fungal CAUTI with bacterial CAUTI in one primary outcome. Concurrent fungal urine growth in patients who also had bacterial CAUTI was retained and reported separately. This approach improves interpretability without reclassifying fungal-only cases as uninfected.

The resistance profile reinforces the need for institution-specific treatment guidance. HLAR, ESBL, MDR/W-OPOR, MBL, VRE, and NDM were common, and 29.9% of bacterial CAUTI patients had at least two recorded resistance phenotypes. Co-occurrence may represent a single multidrug-resistant organism, multiple organisms in the same patient, or hierarchical coding of one mechanism. OXA-48 and MDR coding were harmonized. The unresolved MRR field was not converted into a MarR molecular finding because the data did not document such testing.

COVID-19 remained independently associated with bacterial CAUTI. Severe SARS-CoV-2 infection often required prolonged ventilation, sedation, immobility, systemic steroids, and complex nursing care [13,17–19]. The present analysis is broader than the prior COVID-specific report and explicitly discloses the overlapping cohort and the revised bacterial-only definition.

This study has several limitations. It was retrospective and single-center; event timing was incomplete; pre-ICU catheter exposure and exact CAUTI onset were unavailable; and residual confounding by illness severity and treatment exposure is likely. Removing ICU length of stay/catheter duration from the primary model avoids conditioning on a potential consequence of CAUTI, but the cumulative-incidence logistic model still cannot account fully for differential time at risk; reliable onset dates would be required for a time-to-event or competing-risk analysis. All patients were catheterized, so the study cannot compare catheterized with non-catheterized patients. ICU length of stay was missing in 44 records, and BMI and admission biomarkers had substantial missingness. A complete APACHE II or SOFA score was unavailable for the full cohort. Blood cultures were not documented as systematic at CAUTI diagnosis, so bacteremic CAUTI could not be classified. Microbiology fields were non-mutually exclusive patient-level indicators rather than a relational isolate-level database. Finally, the observed mortality difference was smaller than the approximate 80% detectable difference and remained compatible with no difference.

Strengths include the large consecutive source cohort, explicit bacterial case definition, complete reanalysis after exclusion of fungal-only cases, detailed laboratory procedures, direct descriptive characterization of observed ICU catheter exposure, person-time normalization of TISS-28, an admission-characteristic model that avoids conditioning on post-admission duration, distribution-sensitive biomarker reporting, resistance co-occurrence analysis, and provision of aggregate microbiology summaries and an accompanying data dictionary.

## Conclusions

Bacterial CAUTI occurred in 9.3% of the revised ICU cohort. Female sex, obesity, and COVID-19 were independently associated in the model restricted to admission characteristics. Patients with bacterial CAUTI had substantially longer observed ICU stay/catheter exposure, but the direction of this relationship could not be determined and no causal effect of duration was estimated. Bacterial CAUTI was not associated with in-hospital death after adjustment for admission characteristics. Person-time normalization showed that much of the apparent intervention burden reflected longer observation rather than uniformly higher daily intensity. Strict catheter stewardship, clear separation of bacterial infection from fungal-only disease or colonization, reproducible microbiological methods, and treatment guided by local resistance patterns remain central to prevention and management.

## Supporting information

S1 File Supporting tables. Table A: All TISS-28 intervention rates normalized to observed ICU days. Table B: Admission-characteristic-adjusted logistic regression for in-hospital death. Table C: Missing data for key variables in the revised primary cohort. Table D: Rare bacterial groups and common resistance-phenotype co-occurrences.
(DOCX)

S1 Data Aggregate microbiology frequencies (301 bacterial CAUTI records). Counts of 1–4 are withheld.
(CSV)

S2 Data Screening summary. (CSV)

S3 Data Data dictionary. (XLSX)

## Abbreviations

BSI: Bacterial bloodstream infection
CAUTI: Catheter-associated urinary tract infection
CFU: Colony-forming units
CI: Confidence interval
COVID-19: Coronavirus disease 2019
CRP: C-reactive protein
ESBL: Extended-spectrum beta-lactamase
HLAR: High-level aminoglycoside resistance
ICU: Intensive Care unit
MBL: Metallo-beta-lactamase
MDR: Multidrug-resistant
NDM: New Delhi metallo-beta-lactamase
OR: Odds ratio
TISS-28: Therapeutic Intervention Scoring System-28
VAP: Ventilator-associated pneumonia
VRE: Vancomycin-resistant Enterococcus
WBC: White blood cell count
