# Supplementary material for: Characteristics and outcomes of ICU patients with bacterial catheter-associated urinary tract infection
Source: PLoS One. 2026 Sep 25;21(9):e0358796. doi: 10.1371/journal.pone.0358796 (PMC13614587; doi:10.1371/journal.pone.0358796)
Supplement: Supplementary file 1 [file pone.0358796.s001.docx]

Supporting information

Characteristics and outcomes of ICU patients with bacterial catheter-associated urinary tract infection

Manuscript ID: PONE-D-26-25442

Table A. All TISS-28 intervention rates normalized to observed ICU days.

| **Intervention** | **No bacterial CAUTI per 100 days** | **Bacterial CAUTI per 100 days** | **Rate ratio (95% CI)** | **p value** |
| --- | --- | --- | --- | --- |
| Monitoring | 99.88 | 99.90 | 1.00 (1.00-1.00) | 0.795 |
| Laboratory blood sampling | 98.72 | 98.76 | 1.00 (0.99-1.01) | 0.952 |
| Single-drug administration | 0.07 | 0.04 | 0.57 (0.16-2.08) | 0.397 |
| Two-drug administration | 15.19 | 0.01 | 0.00 (0.00-0.00) | <0.001 |
| Multiple-drug administration | 99.55 | 99.76 | 1.00 (1.00-1.00) | 0.046 |
| Standard dressing care | 83.32 | 98.30 | 1.18 (1.15-1.21) | <0.001 |
| Frequent dressing changes | 3.78 | 0.93 | 0.25 (0.13-0.48) | <0.001 |
| Drain care | 21.32 | 30.55 | 1.43 (1.18-1.75) | <0.001 |
| Mechanical ventilation | 79.05 | 81.21 | 1.03 (0.99-1.06) | 0.136 |
| HFNC or CPAP respiratory support | 8.47 | 5.60 | 0.66 (0.44-0.99) | 0.045 |
| Respiratory support without mechanical ventilation | 45.15 | 37.79 | 0.84 (0.72-0.98) | 0.026 |
| Artificial airway care | 91.60 | 95.35 | 1.04 (1.02-1.06) | <0.001 |
| Respiratory physiotherapy | 57.40 | 69.72 | 1.21 (1.10-1.35) | <0.001 |
| Single vasoactive drug | 43.39 | 45.79 | 1.06 (0.96-1.16) | 0.249 |
| Multiple vasoactive drugs | 33.41 | 21.16 | 0.63 (0.53-0.75) | <0.001 |
| Massive fluid loss/high-volume fluid administration | 9.90 | 4.22 | 0.43 (0.31-0.58) | <0.001 |
| Arterial catheter | 97.19 | 97.44 | 1.00 (0.99-1.01) | 0.647 |
| Pulmonary artery catheter | 0.02 | 0.01 | 0.44 (0.05-3.71) | 0.453 |
| Central venous catheter | 96.45 | 97.90 | 1.02 (1.00-1.03) | 0.085 |
| Cardiopulmonary resuscitation | 0.88 | 0.38 | 0.43 (0.30-0.62) | <0.001 |
| Calibrated minimally invasive hemodynamic monitoring | 1.69 | 2.10 | 1.25 (0.67-2.33) | 0.493 |
| Noncalibrated minimally invasive hemodynamic monitoring | 0.01 | 0.00 | Not estimable | NA |
| Renal replacement therapy | 13.70 | 12.90 | 0.94 (0.71-1.25) | 0.672 |
| Quantitative urine-output monitoring | 98.88 | 99.24 | 1.00 (1.00-1.01) | 0.277 |
| Forced diuresis | 74.11 | 78.05 | 1.05 (1.00-1.11) | 0.040 |
| Dialysis catheter insertion | 1.23 | 1.11 | 0.90 (0.70-1.15) | 0.395 |
| Treatment of acidosis/alkalosis | 3.69 | 1.82 | 0.49 (0.37-0.67) | <0.001 |
| Parenteral nutrition | 45.00 | 37.95 | 0.84 (0.73-0.98) | 0.024 |
| Enteral nutrition | 68.29 | 73.71 | 1.08 (1.02-1.14) | 0.006 |
| Single ICU intervention | 6.73 | 5.75 | 0.85 (0.76-0.96) | 0.006 |
| Multiple ICU interventions | 0.86 | 0.57 | 0.66 (0.49-0.91) | 0.010 |
| Procedures outside the ICU | 4.92 | 4.42 | 0.90 (0.73-1.10) | 0.302 |
| Intracranial pressure monitoring | 0.17 | 0.18 | 1.06 (0.58-1.95) | 0.843 |

Rates are aggregate intervention-days per 100 TISS observation-days. Poisson models used log(observation days) as an offset and robust standard errors. Rate ratios were not estimated when either group had zero recorded intervention-days.

Table B. Admission-characteristic-adjusted logistic regression for in-hospital death.

| **Covariate** | **Adjusted OR** | **95% CI** | **p value** |
| --- | --- | --- | --- |
| Bacterial CAUTI | 0.95 | 0.75-1.22 | 0.708 |
| Age, per 10 years | 1.21 | 1.15-1.26 | <0.001 |
| Female sex | 1.03 | 0.89-1.20 | 0.655 |
| Obesity | 1.02 | 0.85-1.23 | 0.822 |
| COVID-19 | 2.21 | 1.74-2.80 | <0.001 |
| Diabetes mellitus | 1.11 | 0.91-1.35 | 0.299 |
| Heart and/or respiratory failure | 1.04 | 0.89-1.22 | 0.639 |
| Atrial fibrillation | 1.52 | 1.21-1.91 | <0.001 |
| Acute myocardial infarction or ischemic stroke | 1.48 | 1.01-2.17 | 0.043 |

Complete-case n=3170; deaths=1487; model AUC=0.624. The model includes bacterial CAUTI and demographic/clinical characteristics available at or before ICU admission. ICU length of stay/catheter duration, TISS-28 intensity, and systemic steroid therapy were not included because they accrued after admission or lacked reliable temporal ordering relative to CAUTI.

Table C. Missing data for key variables in the revised primary cohort.

| **Variable** | **Missing n** | **Missing %** |
| --- | --- | --- |
| Age | 61 | 1.9 |
| BMI | 1286 | 39.8 |
| ICU length of stay | 44 | 1.4 |
| Observed ICU catheter exposure | 44 | 1.4 |
| TISS observation days | 12 | 0.4 |
| CRP at admission | 1402 | 43.4 |
| WBC at admission | 867 | 26.8 |
| Procalcitonin at admission | 1604 | 49.6 |
| Glucose at admission | 1977 | 61.2 |
| Lactate at admission | 1980 | 61.3 |

In the individual-level clinical source dataset, blank cells denote missing values. Complete-case analysis was used for the multivariable models.

Table D. Rare bacterial groups and common resistance-phenotype co-occurrences.

| **Rare bacterial group** | **n (%)** | **Resistance phenotype pair** | **n (%)** |
| --- | --- | --- | --- |
| Citrobacter | 8 (2.7) | MBL + NDM | 23 (7.6) |
| Haemophilus | 7 (2.3) | MDR/W-OPOR + MBL | 16 (5.3) |
| Morganella | 6 (2.0) | HLAR + ESBL | 15 (5.0) |
| Achromobacter | 1 (0.3) | ESBL + MDR/W-OPOR | 14 (4.7) |
| Raoultella | 1 (0.3) | MDR/W-OPOR + VRE | 11 (3.7) |
|  |  | HLAR + MBL | 9 (3.0) |
|  |  | ESBL + MBL | 8 (2.7) |
|  |  | HLAR + MDR/W-OPOR | 8 (2.7) |
|  |  | MBL + VRE | 7 (2.3) |
|  |  | ESBL + VRE | 6 (2.0) |
|  |  | HLAR + VRE | 6 (2.0) |
|  |  | MRSA + MLSBK | 6 (2.0) |

Variables are patient-level and non-mutually exclusive. Percentages use 301 bacterial CAUTI records as denominator. NDM is an MBL subtype; NDM-MBL co-recording partly reflects hierarchical coding. Concurrent fungal urine growth was recorded in 54/301 (17.9%) bacterial CAUTI patients.
